# *Triatoma maculata* colonises urban domicilies in Boa Vista, Roraima, Brazil

**DOI:** 10.1590/0074-02760160026

**Published:** 2016-10-13

**Authors:** Alice Ricardo-Silva, Teresa Cristina Monte Gonçalves, José Francisco Luitgards-Moura, Catarina Macedo Lopes, Silvano Pedrosa da Silva, Amanda Queiroz Bastos, Nathalia Coelho Vargas, Maria-Rosa Goreti Freitas

**Affiliations:** 1 Fundação Oswaldo Cruz, Instituto Oswaldo Cruz, Laboratório Interdisciplinar de Vigilância Entomológica em Diptera e Hemiptera, Rio de Janeiro, RJ, Brasil; 2 Universidade Federal de Roraima, Núcleo Observatório de Saúde de Roraima-ObservaRR, Boa Vista, RR, Brasil; 3 Secretaria de Saúde, Núcleo Estadual de Entomologia, Boa Vista, RR, Brasil; 4 Núcleo de Entomologia do Distrito Sanitário Especial Indígena Yanomami, Boa Vista, RR, Brasil; 5 Fundação Oswaldo Cruz, Instituto Oswaldo Cruz, Laboratório de Mosquitos Transmissores de Hematozoários, Rio de Janeiro, RJ, Brasil

**Keywords:** Chagas disease, dispersal, artificial ecotope

## Abstract

During a medical entomology course in Boa Vista, Roraima, colonies of *Triatoma maculata* closely associated with pigeon nests were observed in concrete air-conditioner box located on the external plastered and cemented walls of a modern brick-built apartment block. In only one eight-hole ceramic brick, located inside one air-conditioner box, 127 specimens of *T. maculata* were collected. *T. maculata* is a recognised vector of *Trypanosoma cruzi* in the surrounding area and its domiciliation increases the risk of Chagas disease transmission.

*Triatoma maculata* has been found infected with *Trypanosoma cruzi* (Lent & Wygodzinsky 1979, Rojas et al. 2008). Usually, *T. maculata* is collected in bird nests, mostly pigeons, and annexes to the human peridomicile such as chicken houses, pigsties and horse corrals (Lent & Wygodzinsky 1979). The geographic distribution of *T. maculata* has been reported as Colombia, Guyana, Suriname, Venezuela and Brazil (in the Roraima state). In Venezuela *T. maculata* is regarded as the second most important vector species in the transmission of Chagas disease, after *Rhodnius prolixus*. *R. prolixus* is related to domiciliated transmission, invasion and reinfestation of houses after control measures in Venezuela (Feliciangeli & Torrealba 1977, González-Brítez et al. 2010, Reyes-Lugo et al. 2011) where oral outbreaks have been reported, mainly related to the presence of sylvatic and peridomicile triatomine species (Rojas et al. 2008, García-Alzate et al. 2014, Alarcón de Noya et al. 2015).

Even though two specimens of *T. maculata* had previously been found inside a sofa in a human habitation in an agricultural settlement in Rorainópolis, Roraima, Brazil, in 2005 (Luitgards-Moura et al. 2005), this is the first report of *T. maculata* observed in great numbers in a well-built human dwelling classically considered as unlikely for triatomine infestation.

During a practical lesson in an Entomology Course conducted in September 2015 in Boa Vista, Roraima, Brazil, we took students to the field to collect triatomines in nature in a block of flats where reports of triatomines had been previously done. Regardless of these previous reports, we were surprised by a massive infestation in this urban block of flats (coordinates 2.846761º, -60.649788º, 84 m, Fig. 1). Boa Vista is the capital of Roraima where 63.4% of the population of the state live (total 505.665 inhabitants, IBGE 2015, Fig. 1). Caçari neighborhood is one of the neighborhoods with the highest income in Boa Vista even though the block of flats where the *T. maculata* was registered was built for public workers of lower-middle class. Caçari is a residential urbanised neighborhood bordered by secondary forest. The block of flats where the triatomines was found is comprised of three buildings. Each one has six ground floor apartments and two floors with eight apartments in a total of 22 apartments. The apartments have at least one air-conditioning niche each. The name of this condominium is being withheld for ethical reasons. Nonetheless, one of us (NV) is from the Health Secretary of Roraima state and therefore the desinfestation procedures will be duly undertaken.

**Fig. 1:**
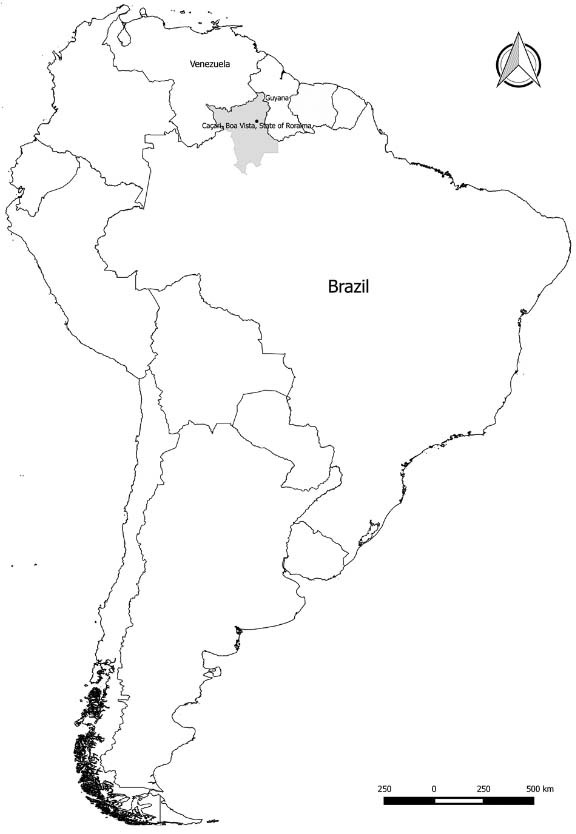
: Brazil, Roraima state, Boa Vista and Caçari neighbourhood where specimens of *Triatoma maculata* were collected, September 2015.

In only one eight-hole ceramic brick, taken from an empty concrete box of an air conditioner (air-conditioning niche) we collected and counted 127 specimens of all stages of *T. maculata*, many fully engorged with blood, possibly from pigeons *Columba livia* that were observed co-inhabiting the air conditioner niche (Fig. 2). Other air-conditioning niches were not investigated due to the fact that the objective of finding triatomines in a practical lesson in the field was fulfilled. The triatomine bugs were identified using dichotomous keys proposed by Lent and Wygodzinsky (1979). *T. maculata* specimens were using the interior of concrete air conditioner niches underneath cement fiber tiled roofs as habitats, in well-built plastered and cemented-walls in a block of flats located in the Caçari neighborhood, Boa Vista, Roraima.

**Fig. 2:**
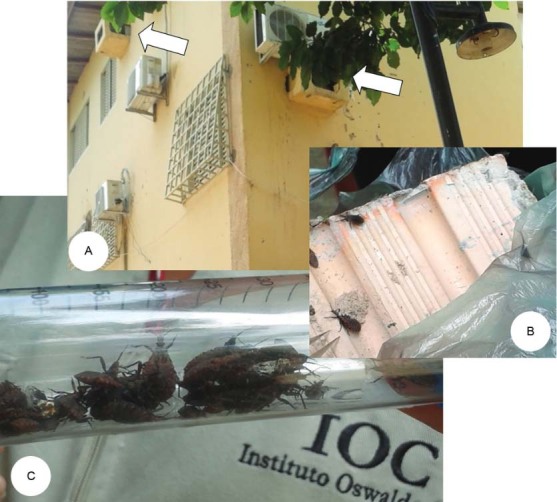
: external concrete-made air conditioner boxes (A, arrows), a eight-hole ceramic brick (B) were *Triatoma maculata* specimens were found (C) in Boa Vista, Roraima, Brazil, September 2015.

All stages of development, from eggs to adults of *T. maculata*, were found in a eight-hole ceramic brick inside a concrete air-conditioner niche in a wall of a building in Boa Vista which characterises an established colonisation process for this triatomine species considered as a *T. cruzi* vector. In a total of 127 specimens a reduced amount of adults were found, no females and only one male, among four nymphs of first instar, 27 nymphs of second instar, 40 nymphs of third instar, 33 nymphs of fourth instar and 21 nymphs of fifth instar. Triatomine vestiges such as feces and exuviae were found inside the holes of the ceramic brick and feces were observed on the building walls and also in the wall were the brick was. After identification, triatomines were frozen at -20ºC. Molecular detection for protozoans is ongoing.

Because September is a hot and dry month in Boa Vista, we believe that the finding of a majority of nymphs is related to adult dispersion in search of new blood sources and high temperatures. The simple fact that the collected specimens were fully engorged of blood does not necessarily means that there were plenty of food sources, since the adults might have dispersed to ensure offspring survival (Lehane et al. 1992).

The fact that *T. maculata* can live in external niches in well plastered walls of the human dwelling is a new fact in the epidemiology of Chagas disease in the Americas that should not be overlooked. The domiciliation of *T. maculata* adds to the synanthropism seen for other hematophagous insects and the associated risks of this co-inhabiting for pathogens transmitted by them. In fact, talking with the individuals living in the condominium, there were other reports of intradomiciliary findings of triatomines as well as complaints about insect biting.

The relevance of this communication is related to the increased risk of transmission of *T. cruzi* and the establishment of Chagas disease in an urban landscape due to proximity of the vector to the human host and the close association with pigeons. A strong indication of a relationship between pigeons and *T. maculata* infestation was observed in the urban area of Boa Vista, Roraima, as previously seen by *T. infestans* in San Juan, Argentina (Vallvé et al. 1996), and Arequipa, Peru (Levy et al. 2014).

Pigeons have become an urban pest worldwide by colonising man made niches such as air-conditioner boxes (there are now many empty boxes as the new split air-conditioner technology makes the use of these boxes redundant), underneath roofs, windows and roof ledges, statues and wherever they find a place where they can rest or make nests. Pigeons can host a plethora of potentially harmful zoonotic pathogens including West Nile virus (Spedicato et al. 2016) and other viruses (Phan et al. 2013), *Chlamydophila psittaci*, *Cryptococcus neoformans* (Haag-Wackernagel & Moch 2004), *Escherichia coli* and *Salmonella enterica* (Morabito et al. 2001, Pedersen et al. 2006, Abulreesh 2011).

The spontaneous reporting on the occurrence of *T. maculata* associated with the presence of pigeons in residences located in other neighbourhoods of Boa Vista (Roraima State Secretary of Health, personal communication) supports the hypothesis that triatomines can accidentally invade houses from those pigeon nests. So, the presence of pigeon nests may represents a risk for the population, as an attractive ecotope for *T. maculata*, where it can build dense populations (Vallvé et al. 1996).

During search for food or for new nesting places pigeons may carry triatomine eggs, nymphs or adults in their feathers (Ribeiro Jr et al. 2006) and help in the dispersal and establishment of new triatomine colonies in a urban scenario. These new colonies of insects may have access to mammals infected with *T. cruzi* in the new environment. During our field work we observed that the air conditioning niches, which are on the outside of the wall, are often separated merely by a piece of cardboard providing a connection between the intradomiciliary space where human individuals reside and peridomiciliary habitats where pigeons and triatomines thrive. Given the recurring reports of triatomine bites by local residents, this is most likely happening already.

The colonisation process is characterised by finding nymphs inhabiting the same ecotope (OMS 1991, Almeida et al. 2015). This might be related to the search of new food sources, or to environmental changes promoted by human actions, such as deforestation. These processes stimulate adult dispersion, which might find a receptive environment in artificial ecotopes (Forattini et al. 1979, Aragão 1983, González-Brítez et al. 2010). In Venezuela this process has been described for *T. maculata* using geometric morphometry to analyse wings and by the variability in the *b*-tubuline marker, coupled with the presence of nymphs inside the houses (García-Alzate et al. 2014). In the present observation in Roraima, we found eggs, nymphs in all development stages and one adult, which is the first encounter of *T. maculata* colonising domiciles in Brazil.

The likely origin of the collected specimens inhabiting air-conditioning niches could be from palm trees in the surroundings even though *T. maculata* has not been registered in urban sites in Boa Vista (Luitgards-Moura et al. 2005). There are chicken houses nearby. The possibility of expansion of the geographical distribution for *T. maculata* through passive carriage by pigeons, the possibility of encountering *T. cruzi* infected animals and the colonisation of the human dwelling are important risk factors for urban vectorial transmission of *T. cruzi* that should be addressed with due care and urgency. This results show the importance of pigeon management aiming at triatomine control.
